# Association Between the Community Prevalence of COVID-19 and Daily Unscheduled Absences of Anesthesiologists, Nurse Anesthetists, and Residents in an Academic Anesthesia Department

**DOI:** 10.7759/cureus.30730

**Published:** 2022-10-26

**Authors:** Franklin Dexter, Richard H Epstein, Anil A Marian

**Affiliations:** 1 Anesthesia, University of Iowa, Iowa City, USA; 2 Anesthesiology, University of Miami Miller School of Medicine, Miami, USA

**Keywords:** anesthesia department management, industrial engineering, operations research, pandemic, covid-19, nurse anesthetist, anesthesiology resident, presenteeism, staff scheduling, unscheduled absences

## Abstract

Introduction

An “unscheduled absence” refers to an occurrence when an employee does not appear for work and the absence was not approved in advance by an authorized supervisor. Daily unscheduled absences need to be forecasted when doing staff scheduling to maintain an acceptable risk of being unable to run all anesthetizing locations and operating rooms planned. The number of extra personnel to be scheduled needs to be at least twice as large as the mean number absent. In an earlier historical cohort study, we found that our department’s modeled risks of being unavailable unexpectedly differed among types of anesthesia practitioners (e.g., anesthesiologists and nurse anesthetists) and among weekdays (i.e., Mondays, Fridays, and workdays adjacent to holidays versus other weekdays). In the current study, with two extra years of data, we examined the effect of the coronavirus COVID-19 pandemic on the frequency of unscheduled absences.

Methods

There were 50 four-week periods studied at a large teaching hospital in the United States, from August 30, 2018 to June 29, 2022. The sample size of 120,687 person-assignment days (i.e., a person assigned to work on a given day) included 322 anesthesia practitioners (86 anesthesiologists, 88 certified registered nurse anesthetists, 99 resident and fellow physicians, and 49 student nurse anesthetists). The community prevalence of COVID‑19 was estimated using the percentage positive among asymptomatic patients tested before surgery and other interventional procedures at the hospital.

Results

Each 1% increase in the prevalence of COVID-19 among asymptomatic patients was associated with a 1.131 increase in the odds of unscheduled absence (P < 0.0001, 99% confidence interval 1.086 to 1.178). Using an alternative model with prevalence categories, unscheduled absences were substantively more common when the COVID-19 prevalence exceeded 2.50%, P $\leqslant$ 0.0002. For example, there was a 1% unscheduled absence rate among anesthesiologists working Mondays and Fridays early in the pandemic when the prevalence of COVID-19 among asymptomatic patients was 1.3%. At a 1% unscheduled absence rate, 67 would be the minimum scheduled to maintain a <5.0% risk for being unable to run all 65 anesthetizing locations. In contrast, there was a 3% unscheduled absence rate among nurse anesthetists working Mondays and Fridays during the Omicron variant surge when the prevalence was 4.5%. At a 3% unscheduled absence rate, 70 would be the minimum scheduled to maintain the same risk of not being able to run 65 rooms.

Conclusions

Increases in the prevalence of COVID-19 asymptomatic tests were associated with more unscheduled absences, with no detected threshold. This quantitative understanding of the impact of communicable diseases on the workforce potentially has broad generalizability to other fields and infectious diseases.

## Introduction

An “unscheduled absence” refers to an occurrence when an employee does not appear for work and the absence was not approved in advance by an authorized supervisor [1]. From the perspective of making operating room assignments for anesthesia practitioners, if approval was not given before the final assignments for the next workday were posted, such absences are counted as unscheduled [2,3]. If, subsequent to such posting, an individual calls in to report they are unable to work because of illness or another reason, this can cause disruption to the operating room schedule, impact timely patient care, and potentially result in additional departmental expenses to cover for the absent anesthesia practitioner.

Estimates for the unscheduled absence rate are needed for an appropriate staff schedule to maintain an acceptable (e.g., 5%) risk of being unable to run all planned anesthetizing locations from having an inadequate number of available practitioners. For example, suppose that, daily, there are 65 operating room and non-operating room locations (e.g., interventional radiology) [3]. The studied department comprises nurse anesthetists, anesthesiology residents and fellows, and student registered nurse anesthetists [3]. Calculations show the number of extra anesthesia practitioners to be scheduled daily to cover unscheduled absences (i.e., how many beyond the number needed for first case starts [4,5]). For example, if the mean unscheduled absence rate were 1.0%, then to reliably have at least 65 practitioners working daily (i.e., with the chance of not being able to cover all locations being less than 5%), at least 67 practitioners need to be scheduled [3]. In contrast, for an absence rate of 3.0% (i.e., attendance percentage of 97.0%), there need to be at least 70 practitioners scheduled daily [3]. The results being dependent on the unscheduled absence rate shows the importance of estimating the absence rate for anesthesia departments [3]. Calculations can be made using a single spreadsheet formula but are not arithmetic [3]. For example, if the unscheduled absence is 3.0% and there are 65 anesthetizing locations to staff, although the mean number of practitioners unexpectedly absent would be two per day, the correct answer is five extra practitioners, not two [3]. There should be five extra scheduled because that number maintains a 5% risk of having insufficient staff to run the planned anesthetizing locations [3].

In August 2018, our department implemented an automatic phone call system for notification of an unscheduled absence. We planned to use the data after two years of service, at which time we expected [2] a sufficient sample size to analyze incidences and to improve modeling [3]. That study was performed, in which we found that the unexpected absence rate differed significantly (P < 0.0001) and substantively (greater than 1%) among groups of anesthesia practitioners (e.g., nurse anesthetists compared to anesthesiology residents and fellows) [3]. Also, there were more unscheduled absences on Mondays, Fridays, and workdays adjacent to a holiday compared to such absences on Tuesdays, Wednesdays, and Thursdays that were not adjacent to a holiday [3].

Although the last quarter studied included the middle of 2020, which corresponded to the start of the coronavirus COVID-19 pandemic, we saw no suggestion of an increased unscheduled absence rate [3]. That was inconsistent with qualitative reports of observed increases in absenteeism among healthcare workers during the early phases of this pandemic [6]. We speculated that not seeing an effect on absenteeism in our department was because Johnson County, where the University of Iowa is located, was minimally impacted during the period of overlap between the COVID-19 pandemic and our previous study [3]. We planned to repeat our evaluation in August 2022 to evaluate the potential effects of the pandemic on absenteeism.

In the current study, we analyzed changes over time in the prevalence of COVID-19 among asymptomatic patients who were tested prior to procedures performed at the hospital. We used that endpoint as a marker for the community prevalence of COVID-19. We evaluated whether increases in the prevalence of asymptomatic COVID-19 were associated with more unscheduled absences of anesthesia practitioners in our department [7]. We also evaluated at what level of prevalence of asymptomatic COVID-19 infection unscheduled absences were sufficiently common to change appropriate managerial decision-making related to staff scheduling.

## Materials and methods

The University of Iowa Institutional Review Board declared on September 8, 2022 that this project (#202209058) does not meet the regulatory definition of human subjects research because the activity is limited to retrospective analyses of deidentified data and is a work-scheduling improvement project. This project was a two-year extension of the earlier project (#202008135) declared on August 12, 2020, not to be human subjects research [3].

Starting on August 6, 2018, the University of Iowa Department of Anesthesia used a dedicated phone number with an automated voice message recording system for anesthesia practitioners to report unplanned absences on regular workdays [3]. This was implemented using Skype for Business (Microsoft, Redmond, WA), with automatic recording of phone messages as an audio file, transcription to text, and forwarding both to a Microsoft Exchange email address. The practitioner provided information including name, role, and assigned location [3]. At 6:00 AM, the audio files were sent automatically to the clinical director of the day [3]. Any notifications recorded after 6:00 AM generated an immediate message to the clinical director of the day [3]. The clinical director listened to each recording, confirmed the practitioner and assigned location, and updated the department’s staff assignment database [3].

The earlier study examined the two-year period from August 12, 2018 to August 8, 2020 [3]. We knew from a study performed using data from the University of Miami that two years would be suitable for comparing groups [2], and that was found [3]. The earlier study showed the results summarized in the examples given in the Introduction and Table 1 [8].

**Table 1:** Examples of unscheduled absence rates on the percentage of days with various numbers of unscheduled absences. a. The probability of identifying > *x* events in *n* independent trials with a probability *p* of the event occurring in an individual trial can be calculated in Excel using the formula 1 - BINOM.DIST(*x*, *n*, *p*, TRUE), or in Google Sheets using 1 - BINOMDIST(*x*, *n*, *p*, TRUE). We performed these calculations using Stata, using the command: display round(100*(1 - binomial (70, 5, 0.03)), 0.01). The 1% unscheduled absence rate is applicable to a 1.3% asymptomatic prevalence [8], and the number of anesthesiologists scheduled Mondays or Fridays. The 2% absence rate is applicable to a 1.3% asymptotic prevalence [8], and the number of nurse anesthetists scheduled Mondays or Fridays. The 3% absence rate is applicable to a 4.5% prevalence, as seen during the Omicron variant surge, and the number of nurse anesthetists scheduled Mondays or Fridays. b. For example, if the unscheduled absence rate in the department is 1.0% and there are 65 anesthetizing locations to be staffed, on 52.03% of days one should expect to have zero unscheduled absences, on 47.97% of days at least one such absence, on 14.14% at least two such absences, etc. c. The extra personnel to be scheduled can be compared to the mean number absent. For a 1.0% unscheduled absence rate, the ratio equals 3.0, where 3.0 = (67-65)/(0.01×67). For a 2.0% rate, the ratio equals 2.2, where 2.2 = (68-65)/(0.02×68). For a 3.0% rate, the ratio equals 2.4, where 2.4 = (70-65)/(0.03×70).

Number of Unscheduled Absences	Unscheduled absence rate 1.0%^a^	Unscheduled absence rate 2.0%	Unscheduled absence rate 3.0%
0 unscheduled absences among 65 scheduled	52.03%^b^	26.90%	13.81%
1 or more unscheduled absences among 65 scheduled	47.97%^b^	73.10%	86.19%
2 or more unscheduled absences among 66 scheduled	14.14%^b^	38.14%	59.26%
3 or more unscheduled absences among 67 scheduled	2.98%^c^	15.06%	32.61%
4 or more unscheduled absences among 68 scheduled		4.75%^c^	14.74%
5 or more unscheduled absences among 69 scheduled			5.63%
6 or more unscheduled absences among 70 scheduled			1.86%^c^

Our statistical analyses were performed using the N = 204,841 combinations of persons and days in the department’s scheduling software program (QGenda, Atlanta, GA), with each combination representing an assignment. We excluded vacations, meetings, post-call, weekends, holidays, clinical assignments without potential operating room cases (e.g., pain medicine), and non-clinical assignments (e.g., department chair in meetings) [3,7]. Unscheduled absences were inferred as days when the practitioner had a clinical assignment in the scheduling system, finalized on the previous working day, but called into the unscheduled absence system to report that they were unable to work that day [3]. After making total counts, we then excluded from analyses the people who were not scheduled for at least five four-week periods during the study interval, because these people would potentially be identifiable from the department’s daily assignment tables, posted on the hospital intranet. As done previously, weekdays were divided into two groups, one group being Tuesdays, Wednesdays, and Thursdays not adjacent to a university holiday and the other group being Mondays, Fridays, or days adjacent to a holiday [2,3]. For example, the Wednesdays before Thanksgiving (i.e., holiday Thursdays) were in the latter group.

Compared with the study of the first two years of data (August 2018 to August 2020) [3], the novel independent variable was the percentage of pre-procedure polymerase chain reaction SARS-CoV-2 tests that were positive among asymptomatic patients during the four-week period. All denominators were at least 2740 tests (i.e., sufficiently large for the percentage itself to be used as a summary measure). Logistic regressions were performed with the dependent variable being the logit of the proportional risk of unscheduled absence. However, for ease of presentation, tables and graphs were created with the dependent variable being the percentage incidences of unscheduled absence rather than the logit. This choice was reasonable because the incidences were much less than 10%, such that the logit differs negligibly from the proportional incidence (i.e., from the number of absences divided by the number of scheduled days). To associate the percentage of unscheduled absences and the percentage of COVID-19 positive tests, quadratic regression was used (Stata 17.0, College Station, TX). If a linear model were suitable, the result would be that the produced figure would have a linear association with the quadratic model. To make tables, percentage incidences also were reported as categories. The categories used were <1.50%, 1.50% to <2.50%, 2.50% to <3.50%, and $\geqslant$3.50%. Observed incidences within the categories are reported. For example, for 2.34%, the largest observed incidence among the four-week periods with the incidence in the category 1.50% to < 2.50%, the category was presented as having an upper range of 2.34%.

Stata was used for inferential tests, all P-values were two-sided, P < 0.01 was treated as statistically significant, and 99% confidence intervals were reported. The effects of the COVID‑19 prevalence, practitioner type, and weekday on unscheduled absences were analyzed using logistic regression. The analyzed population included 322 anesthesia practitioners (86 anesthesiologists, 88 certified registered nurse anesthetists, 99 residents and fellow physicians, and 49 student nurse anesthetists) (Table 2). Robust variance estimation was used with adjustment for these 322 clusters (STATA 16.1, College Station, TX). Logistic regression results for prevalence as a continuous variable were reported to multiple digits because the units are per 1%. Predictive margins were calculated from the logistic regression model.

**Table 2:** Observed unscheduled absences among groups of anesthesia practitioners. Data are reported as the unscheduled absence percentage (count of unscheduled absences/person-assignment days). “CRNAs” refers to Certified Registered Nurse Anesthetists. The sum of the denominators in the “Anesthesiologists” column and the “Anesthesia providers” column equals the total analyzed (N = 120,687). The “Weekday category” column’s first three rows have Tuesdays, Wednesdays, and Thursdays excluding those adjacent to a federal holiday (e.g., the Tuesday adjacent to the United States Labor Day holiday). The fourth through sixth rows have Mondays, Fridays, and days adjacent to a federal holiday. The “COVID-19 asymptomatic prevalence” column refers to the rate of COVID-19 positive tests among asymptomatic patients tested preoperatively at the hospital before anesthetics and other surgical procedures. The “Anesthesia provider” column includes the combination of the “CRNAs” (N=88), “Residents and fellows” (N=99), and student registered nurse anesthetists (N=49).

Weekday category	COVID-19 asymptomatic prevalence	Anesthesiologists	CRNAs	Residents and fellows	Anesthesia providers
Tue, Wed, Thu, not adjacent to holiday	0%	0.89% (79/8841)	1.19% (121/10179)	0.29% (23/7826)	0.85% (172/20279)
	0.45% to 2.34%	0.76% (73/9559)	1.47% (186/12650)	0.26% (22/8302)	1.07% (250/23444)
	2.77% to 8.48%	1.25% (29/2314)	2.88% (92/3193)	0.54% (11/2024)	2.01% (117/5828)
Mon, Fri, or adjacent to holiday	0%	0.91% (60/6601)	1.84% (140/7605)	0.55% (31/5626)	1.33% (198/14862)
	0.45% to 2.34%	1.15% (77/6673)	1.90% (165/8668)	0.47% (28/5908)	1.37% (226/16484)
	2.77% to 8.48%	2.40% (40/1665)	2.86% (64/2235)	0.68% (10/1460)	2.13% (88/4137)

## Results

The total population of 121,134 operating room clinical assignment days of 360 people (“person-assignment days”) had 1,409 unscheduled absences (1.16%). Analyzed data excluded 447 person-assignment days among 38 people, who, collectively, had no (zero) unplanned sick days. The resulting 120,687 person-assignment days of 322 people had 1,409 unscheduled absences (1.17%) (Table 2). The 322 practitioners each accounted for <1.85% of unscheduled absences.

**Figure 1:**
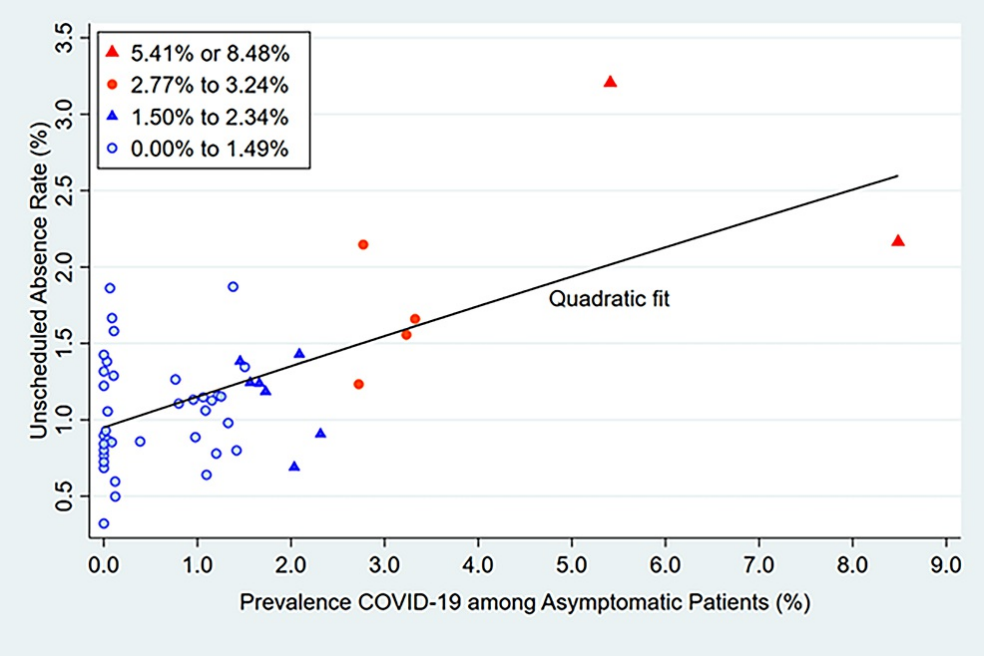
Prevalence of COVID-19 among asymptomatic patients and the unscheduled absence rate among all types of anesthesia practitioners. The categories are <1.50%, 1.50% to <2.50%, 2.50% to <3.50%, and ≥3.50%. The ranges displayed in the legend are the observed prevalence among four-week periods within each category. The Pearson correlation coefficient equals 0.62 (P < 0.0001). The Spearman correlation coefficient equals 0.42 (P = 0.0026).

**Figure 2:**
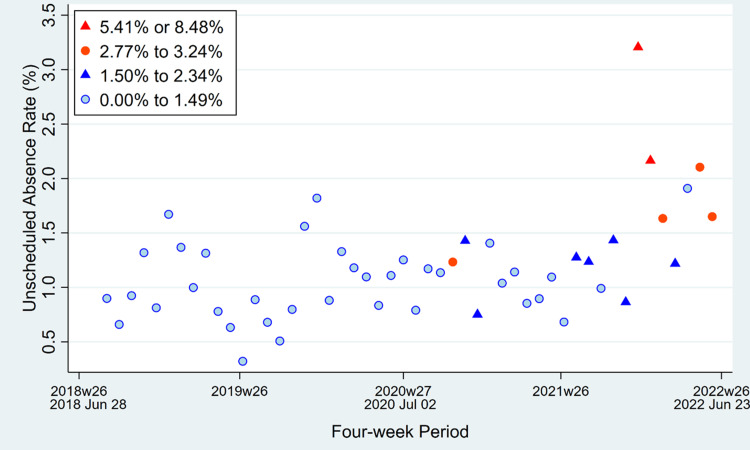
Four-week period and the unscheduled absence rate of all types of anesthesia practitioners, plotted with categories of the prevalence of COVID-19 among asymptomatic patients tested before surgery and other procedures. The categories are <1.50%, 1.50% to <2.50%, 2.50% to <3.50%, and ≥3.50%. The ranges displayed in the legend are the observed prevalence among four-week periods for each category. The notation in the x-axis such as 2018w26 refers to the start of the 26th week in 2018.

An increasing prevalence of COVID-19 among asymptomatic patients scheduled for surgery was associated with more observed unscheduled absences (Table 2). Figure 1 shows that the association reasonably could be treated as linear. Figure 2 shows a lack of interaction between the prevalence rate and the four-week period. For example, if greater prevalence were progressively associated with lesser increases in unscheduled absence rates due to more people receiving a COVID-19 vaccine, Figure 2 would not show the progressive increase over time in unscheduled absences.

Based on Figure 1, the primary logistic regression model treated the prevalence of COVID-19 among asymptomatic patients as a continuous variable. Each 1.0% increase in the prevalence of COVID-19 among asymptomatic patients was associated with a 1.131 increase in odds of unscheduled absence (P < 0.0001, 99% confidence interval 1.086 to 1.178, Table 3 model 1). Using a model with categories of prevalence that match Figure 1, the effect was substantively large when the prevalence exceeded 2.50% (Table 3 model 2). No interactions changed these conclusions (Table 3 models 3 to 5).

**Table 3:** Multivariable logistic regression models examining the association between the prevalence of COVID-19 among asymptomatic patients and unscheduled absences among anesthesia practitioners. “Prevalence COVID-19 (%)” refers to the prevalence of COVID-19 among asymptomatic patients tested preoperatively before surgery and procedures with anesthesia. “Weekday Mon, Fri, or holiday” refers to a weekday Monday, Friday, or a workday adjacent to a holiday, with the reference group being Tuesdays, Wednesdays, or Thursdays, not adjacent to a holiday. Listed anesthesia practitioner groups have reference groups of anesthesiologists. “Prevalence” as a category uses as its reference group a prevalence of 0% to 1.49%. For example, the model in the first five rows has the dependent variable unscheduled absence or not, and independent variables of the prevalence of COVID-19, weekday category, and anesthesia practitioner group.

Model	Coefficient	Estimated odds ratio	P-value	99% confidence interval
1	Prevalence COVID-19 (per 1%)	1.131	<0.0001	1.086 to 1.178
	Weekday Mon, Fri, or holiday	1.34	<0.0001	1.17 to 1.53
	Nurse anesthetists	1.71	0.0004	1.16 to 2.53
	Residents and fellows	0.40	<0.0001	0.24 to 0.65
	Student nurse anesthetists	1.67	0.0069	1.02 to 2.72
2	Prevalence COVID-19 from 1.50% to 2.34%	1.12	0.24	0.87 to 1.44
	Prevalence COVID-19 from 2.77% to 3.24%	4.31	0.0002	1.20 to 2.05
	Prevalence COVID-19 from 5.41% to 8.48%	8.25	<0.0001	1.88 to 3.34
	Weekday Mon, Fri, or holiday	1.34	<0.0001	1.17 to 1.52
	Nurse anesthetists	1.72	0.0003	1.16 to 2.54
	Residents and fellows	0.40	<0.0001	0.24 to 0.65
	Student nurse anesthetists	1.67	0.0065	1.03 to 2.72
3	Prevalence COVID-19 from 1.50% to 2.34%	1.14	0.29	0.83 to 1.56
	Prevalence COVID-19 from 2.77% to 3.24%	1.67	0.0005	1.21to 2.31
	Prevalence COVID-19 from 5.41% to 8.48%	2.62	<0.0001	1.80 to 3.81
	Weekday Mon, Fri, or holiday	1.37	<0.0001	1.17 to 1.61
	Nurse anesthetists	1.72	0.0003	1.16 to 2.54
	Residents and fellows	0.40	<0.0001	0.24 to 0.65
	Student nurse anesthetists	1.67	0.0065	1.03 to 2.72
	Prevalence COVID-19 from 1.50% to 2.34% × Weekday Mon, Fri, or holiday	0.97	0.84	0.66 to 1.43
	Prevalence COVID-19 from 2.77% to 3.24% × Weekday Mon, Fri, or holiday	0.87	0.43	0.56 to 1.36
	Prevalence COVID-19 from 5.41% to 8.48% × Weekday Mon, Fri, or holiday	0.91	0.63	0.56 to 1.48
4	Prevalence COVID-19 from 1.50% to 2.34%	0.91	0.66	0.53 to 1.56
	Prevalence COVID-19 from 2.77% to 3.24%	1.88	0.0040	1.07 to 3.32
	Prevalence COVID-19 from 5.41% to 8.48%	1.92	0.011	0.99 to 3.70
	Weekday Mon, Fri, or holiday	1.34	<0.0001	1.17 to 1.52
	Nurse anesthetists	1.62	0.0024	1.08 to 2.45
	Residents and fellows	0.41	0.0003	0.23 to 0.71
	Student nurse anesthetists	1.75	0.0088	1.01 to 3.04
	Prevalence COVID-19 from 1.50% to 2.34% × Nurse anesthetists	1.45	0.12	0.78 to 2.70
	Prevalence COVID-19 from 1.50% to 2.34% × Residents and fellows	1.01	0.98	0.37 to 2.77
	Prevalence COVID-19 from 1.50% to 2.34% × Student nurse anesthetists	0.92	0.83	0.34 to 2.50
	Prevalence COVID-19 from 2.77% to 3.24% × Nurse anesthetists	0.80	0.39	0.42 to 1.55
	Prevalence COVID-19 from 2.77% to 3.24% × Residents and fellows	0.65	0.29	0.23 to 1.85
	Prevalence COVID-19 from 2.77% to 3.24% × Student nurse anesthetists	0.77	0.52	0.27 to 2.16
	Prevalence COVID-19 from 5.41% to 8.48% × Nurse anesthetists	1.54	0.13	0.73 to 3.24
	Prevalence COVID-19 from 5.41% to 8.48% × Residents and fellows	1.24	0.65	0.37 to 4.19
	Prevalence COVID-19 from 5.41% to 8.48% × Student nurse anesthetists	1.03	0.94	0.38 to 2.83
5	Prevalence COVID-19 (per 1%)	1.131	<0.0001	1.086 to 1.178
	Weekday Mon, Fri, or holiday	1.36	0.0013	1.06 to 1.74
	Nurse anesthetists	1.75	0.0005	1.16 to 2.64
	Residents and fellows	0.35	<0.0001	0.20 to 0.61
	Student nurse anesthetists	1.78	0.0047	1.05 to 3.02
	Weekday Mon, Fri, or holiday × Nurse anesthetists	0.96	0.75	0.71 to 1.31
	Weekday Mon, Fri, or holiday × Residents and fellows	1.27	0.21	0.78 to 2.05
	Weekday Mon, Fri, or holiday × Student nurse anesthetists	0.87	0.44	0.55 to 1.38

The primary logistic regression model (Table 3 model 1) was then applied. The predicted percentage of unscheduled absences was 1% among anesthesiologists working Mondays and Fridays early in the pandemic when the prevalence of COVID-19 among asymptomatic patients was 1.3% [8]. At a 1% incidence of unscheduled absences, 67 practitioners would be the minimum scheduled to assure <5.0% risk for being unable to run all 65 anesthetizing locations (Table 1). In contrast, the predicted percentage of unscheduled absences was 3% among nurse anesthetists working Mondays and Fridays during the Omicron variant surge when the prevalence was 4.5%. At a 3% incidence of unscheduled absences, 70 would be the minimum number of practitioners scheduled to achieve the same risk of not being able to run 65 rooms (Table 1).

## Discussion

We observed a linear association between the prevalence of COVID-19 among the hospital’s asymptomatic surgical patients and the daily number of unscheduled absences among anesthesia practitioners. When the asymptomatic COVID-19 prevalence exceeded 2.5%, the unscheduled absences were substantively more common, sufficient to change how many anesthesia practitioners should be scheduled to work daily (Figures 1, 2). The importance of understanding patterns of unscheduled absences is highlighted by the title of one of the papers by Pandit: “Why are there local shortfalls in anesthesia consultant staffing?” [9]. Time away (“leave,” whether for vacation, study, or unexpected illnesses) needs to be forecasted when choosing the total number of employees and their staff scheduling [9]. Decision-making based on assuming that people will not get sick or have unexpected absences is unrealistic and counterproductive. Even when managers specify risks that they perceive are the maximum they can tolerate organizationally, their decisions are suboptimal unless they apply the appropriate mathematics [10-12].

As Table 1 shows, if you schedule extra practitioners daily to maintain a low risk of being unable to run all anesthetizing locations, the consequence will be that on most days, there will be extra practitioners. This cannot be avoided because the time of day when the first cases begin is the busiest [4], meaning that the maximum number of people is needed at the start of the workday. We previously reviewed how best to use extra personnel on the day of surgery to achieve various objectives in a sequence of priority (e.g., reducing overutilized time) [13]. Such results are relevant to hospitals (such as the one studied) where there are no anesthesia practitioners available to work on a per diem basis on short notice. However, there are practices, especially in large metropolitan areas, where the total number of anesthesiologists or nurse anesthetists available for such scheduling are substantial [14]. Earlier, Rath showed the best way to contract and pay while achieving the optimal balance between being called to work versus not being used [14].

We assured that in the current study, among the studied practitioners, each accounted for <1.85% of unscheduled absences. Thus, it was not the situation that the distribution of unscheduled absences among anesthesia practitioners was highly skewed. Intuitively, some individuals may have a greater chance of becoming infected with a community pathogen than others. For example, while some may choose when not working to remain mostly at home alone, others may have multiple young children and extensive outside activities. Such heterogeneity cannot be examined with available data given that we previously showed the need to have many years of unchanging data to compare individuals [4]. There are no such unchanging data because there was a pandemic.

Our study was a planned reanalysis of our study conducted two years ago [3]. In the more recent data, there were no changes in the findings that nurse anesthetists had a greater prevalence of unscheduled absences than anesthesiologists (Tables 2, 3), and that residents and fellows had a lesser prevalence than the anesthesiologists (Tables 2, 3) [3]. We emphasized previously, and we reemphasize, that in no way should these findings be interpreted as a criticism or commendation of any group [3]. The lower prevalence of unscheduled absences among anesthesiologists or residents and fellows could be a consequence of their working when they are sick (referred to scientifically as “presenteeism”) [15]. What we can conclude from Table 3 is that there was no significant interaction with COVID-19 (i.e., no suggestion that the patterns by type of practitioner were influenced by the pandemic).

Our study was limited in having insufficient data to know how many days people are unavailable for work when an unscheduled absence occurs [3]. One reason is that we do not know the causes of unscheduled absences (e.g., non-medical). A second reason is that many clinicians in our department work >10-hour shifts for fewer than five days per week, provide clinical services at non-operating room locations, or have educational and managerial roles [7]. Therefore, illnesses extending more than one day often do not result in greater than one day absent from providing operating room care, because the practitioners did not have successive days scheduled for operating room care [3]. In addition, if the illness was expected to last for multiple days (e.g., a COVID-19 infection occurred [16]), the days subsequent to the unscheduled abscess may have been approved in advance.

## Conclusions

Anesthesia clinical directors need to judge what percentage of anesthesia residents can be expected in operating rooms daily. They need to judge what percentage of the nurse anesthetists will likely have an unscheduled absence. They similarly need to do the same for anesthesiologists and student nurse anesthetists. (The same applies to other specialties with large numbers of daily scheduled practitioners, such as emergency medicine.) We modeled each group, detected significant differences, and showed that the differences were sufficiently large to affect the number of practitioners to schedule daily. Increases in the prevalence of COVID-19 asymptomatic tests were associated with more unscheduled absences, with no detected threshold. This quantitative understanding of the impact of communicable diseases on the workforce potentially has broad generalizability to other fields where practitioners are scheduled to take care of individual patients and for highly communicable infectious diseases other than COVID-19.
